# Interventions to improve children's access to mental health care: a systematic review and meta-analysis

**DOI:** 10.1017/S2045796019000544

**Published:** 2019-10-17

**Authors:** L. Werlen, D. Gjukaj, M. Mohler-Kuo, M.A. Puhan

**Affiliations:** 1 Epidemiology, Biostatistics and Prevention Institute, University of Zurich, Zurich, Switzerland; 2 La Source, School of Nursing Sciences, HES-SO University of Applied Sciences and Arts of Western Switzerland, Lausanne, Switzerland; 3 Department of Child and Adolescent Psychiatry and Psychotherapy, University Hospital of Psychiatry Zurich, University of Zurich, Zurich, Switzerland

**Keywords:** Children and adolescents, child psychiatry, children, health service research, mental health, pediatrics, systematic reviews

## Abstract

**Aims:**

Mental disorders in children are a significant and growing cause of morbidity worldwide. Although interventions to help overcome barriers along the pathway to accessing health care for children with mental disorders exist, there is no overview of randomised controlled trials (RCTs) on these interventions as yet. This study aimed to systematically identify RCTs of interventions to improve access to mental health care for children and synthesise them using a conceptual framework of access to health care.

**Methods:**

This systematic review was performed following a predefined protocol registered with PROSPERO (ID: CRD42018081714). We searched the databases MEDLINE, EMBASE, PsycINFO and CENTRAL for RCTs up to 15 May 2019 using terms related to the concepts ‘young people,’ ‘mental disorders’ and ‘help-seeking interventions’ and scanned reference lists from relevant studies. Two reviewers independently screened all identified articles in a two-stage process, extracted results on outcomes of interest (knowledge, attitudes, intentions, help-seeking, accessing care, mental health outcomes and satisfaction), assessed the risk of bias and conducted meta-analyses where deemed appropriate.

**Results:**

After screening 5641 identified articles, 34 RCTs were eligible for inclusion. Eighty per cent of universal school-based interventions measuring knowledge (*n* = 5) and 67% measuring attitudes (*n* = 6) reported significantly better results compared with controls on those outcomes, whereas 20% measuring access to care (*n* = 5) and none measuring mental health outcomes (*n* = 7) did. In contrast, 71% of interventions targeting at-risk individuals (*n* = 21) reported better access to care compared with controls, while just 33% (*n* = 6) did for mental health outcomes. For satisfaction with care, this proportion was 80% (*n* = 5). Meta-analyses of interventions measuring initial appointment attendance yielded combined odds ratios of 3.11 (2.07–4.67) for appointment reminder interventions and 3.51 (2.02–6.11) for treatment engagement interventions. The outcomes for universal school-based interventions were heterogeneous and could not be summarised quantitatively through meta-analysis.

**Conclusions:**

To have a population-level effect on improving children's access to mental health care, two-stage interventions that identify those in need and then engage them in the health-care system may be necessary. We need more evidence on interventions to target contextual factors such as affordability and infrastructural barriers.

## Introduction

Mental disorders are one of the most significant causes of disability-adjusted life-years worldwide, and they continue to grow in importance as a major contributor to the global burden of disease (GBD 2015 DALYs and HALE Collaborators, 2016). Because mental disorders usually first occur early in life (Kessler *et al*., 2005) and are characterised by recurrent episodes and symptoms that strongly affect work capacity (Simon *et al*., 2001), they have a significant impact on public health and society.

Childhood and adolescence are particularly critical periods for the identification and treatment of mental disorders. At 45% of the overall burden of disease in 15–19 year-olds, mental health issues are the leading cause of disability in adolescents (The Lancet, 2017). In addition, young patients with a mental disorder have a lower probability of receiving treatment and a longer delay between disease onset and first treatment compared with adults (Christiana *et al*., 2000; Wang *et al*., 2005; Iza *et al*., 2013). Despite the magnitude and importance of mental health problems in childhood and adolescence, international studies have consistently revealed a treatment gap: estimates of the gap between those in need of mental health care and those who access it exceed 50% (Saxena *et al*., 2007).

Levesque *et al*. (2013) define access to health care as ‘the opportunity to reach and obtain appropriate health-care services in situations of perceived need for care’ (Levesque *et al*., 2013). They have proposed a comprehensive conceptual framework describing accessing health care as a series of steps beginning with the opportunity to perceive health-care needs that can lead to opportunities to seek health care, reach health-care services, utilise health-care services and ultimately have health-care needs fulfilled (Levesque *et al*., 2013). At each stage, supply-side dimensions of accessibility of services (e.g., approachability, availability or affordability) interact with demand-side abilities of persons (e.g., abilities to perceive, pay or engage) to determine access to health care (see Appendix 1) (Levesque *et al*., 2013). In other words, the care that is obtained depends on the interplay of characteristics of individuals, such as their socio-economic status or where they live, and those of services and the environment, such as how much services cost and where they are located. Potential barriers that could explain the treatment gap can be found at each transition from step to step along this pathway to accessing care (Levesque *et al*., 2013). Barriers to mental health help-seeking in young people include lack of knowledge about services and stigma about mental health problems (Gulliver *et al*., 2010). As an example of barriers on the supply side, paediatricians perceive a wide variety of organizational hindrances, including inadequate reimbursement and lack of time and space, and many feel they lack the training and confidence to treat mental disorders (Horwitz *et al*., 2007).

To close the treatment gap, interventions targeting one or more dimensions of accessibility of services and/or abilities of persons have been designed to address the barriers along the pathway to accessing care (e.g., screenings, health literacy promotion); however, there is little high-quality evidence on these interventions (National Institute for Health and Clinical Excellence, 2011). Moreover, systematic reviews conducted in the past on interventions to improve access to mental health care for children and adolescents have limited searches to specific types of interventions and disorders (Ingoldsby, 2010; Gulliver *et al*., 2012; Lindsey *et al*., 2014; Anderson *et al*., 2017; Dunne *et al*., 2017; Richardson *et al*., 2017). This study thus aimed to systematically identify randomised controlled trials (RCTs) of all interventions designed to improve access to mental health care for children along the entire pathway to accessing care, describe them using Levesque *et al*. (2013)'s conceptual framework of access to health care (Levesque *et al*., 2013) and conduct meta-analyses for intervention types with comparable outcomes.

## Methods

The methods used for this systematic review are based on the Centre for Reviews and Dissemination's guidance for undertaking reviews in health care (Centre for Reviews and Dissemination, 2009), and our reporting follows the Preferred Reporting Items for Systematic Reviews and Meta-Analysis (PRISMA) (Moher *et al*., 2009). A PRISMA checklist can be found in Appendix 2. We registered our systematic review protocol with the International Prospective Register of Systematic Reviews (PROSPERO, ID: CRD42018081714).

### Types of participants

We included interventions designed for children and adolescents <19 years old, both from the general population and vulnerable groups. If the age range exceeded 18 years old, the intervention was only included if more than 50% of the ages considered were under 19. Interventions that addressed the following disorders from the International Classification of Diseases, 10th Revision (ICD-10) (World Health Organization, 1992) as well as suicidal ideation were considered: F10–F59 and F90–99 (all mental disorders except for mental disorders due to known physiological conditions, disorders of adult personality and behaviour, intellectual disabilities and pervasive and specific developmental disorders including autism spectrum disorders). We also included studies that targeted children with emotional or behavioural problems since children are not always given a specific diagnosis.

### Types of interventions

Any intervention designed to improve access to mental health was included; thus, the intervention could target any one of the five supply-side dimensions or five demand-side abilities included within the conceptual framework. Examples of specific intervention targets are listed next to each dimension or ability in Appendix 1. For example, an intervention could change where services are offered or deliver services via the Internet (National Institute for Health and Clinical Excellence, 2011). The interventions could target the child or adolescent directly or others, including parents/caregivers, teachers, friends or health-care professionals (potential helpers).

### Types of outcome measures

We defined outcomes using the conceptual framework and expanded upon these using conceptualisations from previous systematic reviews on help-seeking and treatment engagement interventions (Gulliver *et al*., 2012; Lindsey *et al*., 2014). Outcomes at all steps in the process of accessing health care were included in the review: knowledge about accessing mental health care, changed attitudes or beliefs about seeking care, intentions to seek care, help-seeking attempts to access health-care services (successful or not) or action taken by a potential helper, mental health outcomes and satisfaction with health-care services. For a study with outcomes on health measures and satisfaction with care to be included in the analysis, the study also had to measure access to care as an outcome. We excluded studies for which it was not possible to calculate any effect sizes.

### Search methods for identification of studies

We performed the literature search on 15 May 2019 in the following electronic databases: MEDLINE, EMBASE, PsycINFO and the Cochrane Central Register of Controlled Trials (CENTRAL). The search strategy included terms relating to the concepts ‘young people,’ ‘mental disorders’ and ‘help-seeking interventions.’ The full search strategy can be found in Appendix 3. Publications not originally published in English were excluded from the search. We enhanced our search by scanning the reference lists of papers (both primary studies and reviews) that were identified by the database search. Duplicates were removed during the title and abstract screening.

### Selection of studies and data extraction

Two reviewers (LW, DG) independently assessed the title and abstract of all identified papers, recorded their decision about whether the paper should be included for full-text assessment and discussed discrepant decisions until a consensus was reached. All papers deemed potentially eligible by the reviewers were included in the full-text assessment, in which the two reviewers decided on study inclusion based on the inclusion criteria and discussed any discrepant decisions until they reached a consensus.

The two reviewers independently extracted data on the following study characteristics: title, first author, year, country, study design, age range, intervention setting, condition in focus, sample size, response rate, intervention condition, control condition, length of intervention, evaluation time points, method of outcome assessment and results.

### Assessment of risk of bias in included studies

Two reviewers (LW, DG) assessed the risk of bias of each article using the Cochrane Collaboration's tool for assessing risk of bias in randomised trials (Higgins *et al*., 2011) and discussed discrepant evaluations until they reached a consensus. Because our review included a large variety of interventions and outcomes, we could rarely assess the heterogeneity, imprecision and indirectness beyond a single or a few studies and therefore decided against using the Grading of Recommendations Assessment, Development and Evaluation (GRADE) approach (Guyatt *et al*., 2011) to judge the overall quality of evidence. Instead, we used the risk of bias assessment as an indicator of the quality of evidence.

### Data synthesis and measures of effect

We mapped the study results using the conceptual framework by Levesque *et al*. (2013) by target of intervention (Fig. 2) and by outcome (Fig. 3). For dichotomous outcomes, we extracted or calculated the odds ratio and 95% confidence interval, whereas, for continuous outcomes, we calculated the standardised mean difference and 95% confidence interval using Cohen's *d* with the package ‘esc’: Effect Size Computation for Meta Analysis in R (Lüdecke, 2017).

For intervention types with comparable outcomes, we conducted meta-analyses using the inverse variance method. We calculated a fixed-effects model if *I*^2^ was <30% and both fixed and random effects models if *I*^2^ was >30% using the package ‘meta’: General Package for Meta-Analysis in R (Schwarzer, 2007).

## Results

### Results of the search and excluded studies

**Fig. 1.**
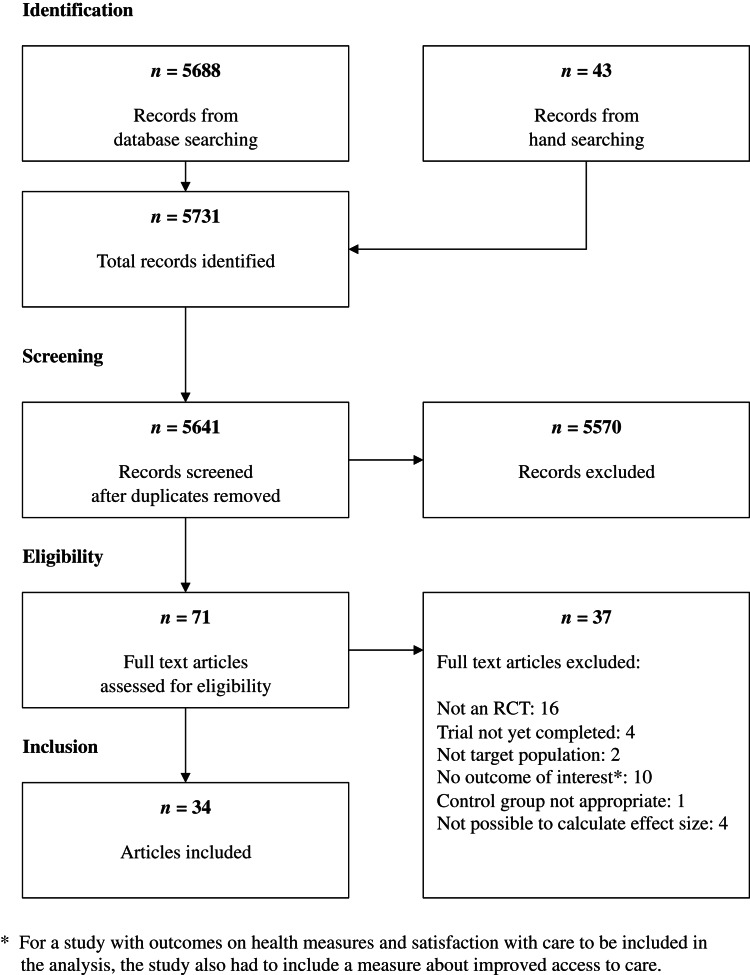
Study flow diagram.

The electronic search yielded 5688 articles, and an additional 43 records were identified through hand searching. Of these 5731 records, 5641 unique studies remained after duplicates were removed. A total of 71 articles were considered eligible for full-text screening following the title and abstract screening. During the full-text screening process, 37 articles were excluded; the full list of articles excluded along with reason for exclusion can be found in Appendix 4. The remaining 34 articles were included in the systematic review. For an overview of the search and screening process, please see the study flow diagram (Fig. 1).

### Included studies

A summary of the characteristics of the 34 RCTs identified through the two-stage screening process can be found in Appendix 5. These studies fell into two main categories: (1) universal school-based interventions targeting the general population (13 studies) and (2) interventions to engage at-risk individuals who had already been identified by the health-care system (21 studies). The vast majority of these studies were conducted in the USA (22 studies); the rest were conducted in Australia (five studies), UK (three studies), Canada (two studies), Portugal (one study) and Israel (one study).

The interventions in the first study category were designed to improve outcomes for general mental health problems, mental distress, suicide, depression and attention-deficit hyperactivity disorder. All studies in the second category took place in health-care settings (e.g., primary care, emergency department, mental health agency) and targeted general mental health problems, behavioural health problems, suicide, depression, substance abuse and conduct disorder. Interventions designed to help younger children access care tended to be addressed towards caretakers, whereas interventions targeting older age groups tended to address the adolescent directly.

**Fig. 2.**
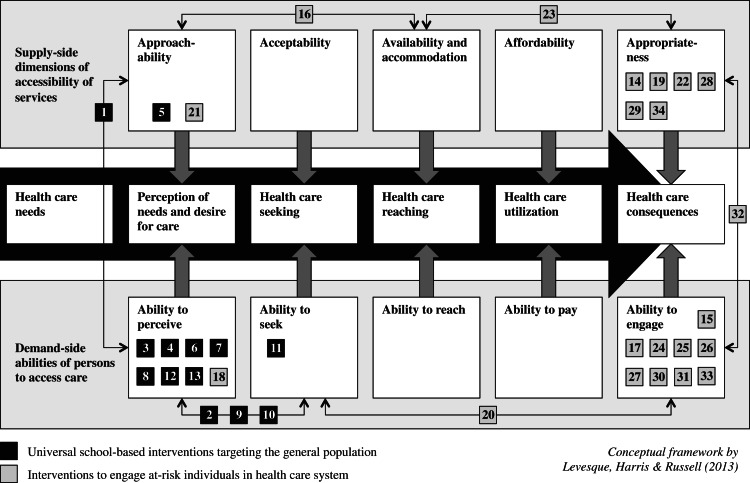
Target of interventions.

Figure 2 provides an overview of the step or steps along the pathway to accessing care that each intervention targeted. Interventions within the first category exclusively targeted service providers' approachability (i.e., service providers making their existence known to individuals) and the abilities of individuals to perceive a need for and to seek care. These interventions included educational curricula, live or virtual contact with a mentally ill person, screenings and helper training programs. The vast majority of engagement interventions from the second category mostly targeted service providers' appropriateness or individuals' ability to engage. Forty-eight per cent (10/21) of these interventions consisted of programs to engage and motivate patients or to improve service providers' communication skills (henceforth called treatment engagement interventions), while 24% (5/21) involved using a telephone or letter reminder mechanism to improve first appointment attendance (henceforth called appointment reminder interventions). Just one intervention was infrastructural in nature and involved providing onsite mental health services for primary care patients. None of the identified studies targeted the acceptability (cultural and social factors that make it possible for individuals to accept services) of service providers, affordability of care, or individuals' personal ability to reach (e.g., their personal mobility or support from their social network) or pay for care.

### Risk of bias in included studies

The results of the risk of bias assessment can be found in Table 1. The randomisation procedure was not described in half of the studies, and two studies described a non-random sequence generation procedure. Details on allocation concealment were only provided in 21% of the studies. In all studies except for one, it was unclear whether a lack of blinding of participants and personnel would influence the outcome. However, we judged that a lack of blinding of outcome assessment would not have an impact on the outcome since most outcomes were evaluated either by questionnaire or service use records. All but one study had a low risk of bias for incomplete outcome data. Three studies did not report all outcomes and three studies had other sources of potential bias.

**Table 1.** Risk of bias of included studies

	a.	b.	c.	d.	e.	f.	g.*
1. Aseltine *et al*. (2007)	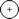	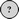	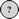	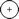	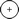	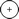	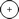
2. Campos *et al*. (2018)	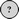	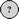	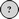	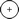	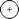	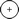	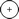
3. Hart *et al*. (2018)	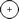	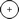	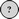	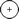	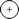	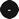	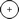
4. Howard *et al*. (2018)	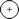	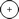	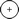	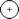	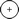	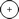	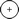
5. Husky *et al*. (2011)	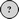	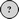	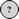	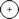	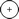	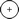	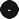
6. Jorm *et al*. (2010)	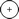	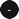	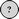	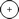	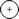	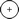	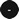
7. Klingman and Hochdorf (1993)	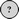	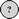	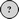	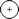	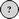	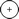	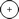
8. Morgan *et al*. (2019)	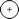	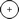	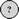	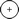	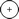	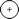	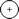
9. Painter *et al*. (2017)	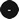	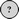	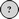	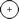	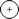	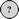	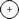
10. Perry *et al*. (2014)	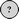	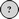	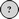	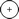	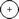	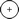	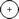
11. Saporito *et al*. (2011)	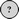	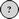	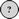	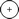	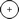	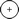	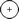
12. Sayal *et al*. (2010)	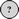	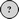	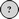	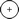	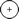	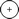	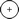
13. Sharpe *et al*. (2017)	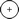	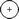	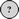	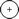	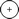	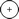	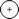
14. Asarnow *et al*. (2005)	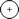	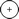	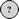	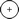	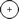	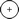	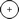
15. Asarnow *et al*. (2011)	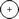	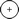	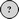	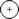	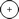	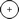	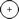
16. Coker *et al*. (2019)	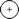	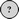	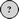	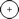	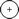	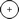	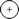
17. Donohue *et al*. (1998)	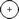	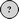	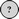	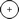	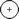	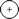	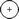
18. Fristad *et al*. (2003)	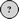	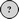	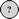	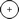	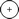	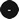	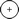
19. Gadomski *et al*. (2010)	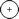	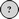	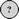	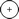	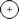	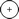	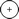
20. Grupp-Phelan *et al*. (2012)	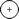	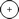	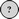	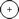	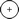	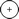	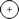
21. Gully *et al*. (2008)	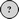	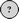	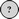	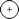	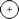	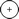	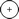
22. Kourany *et al*. (1990)	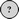	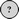	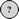	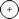	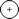	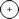	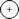
23. Lieberman *et al*. (2006)	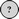	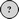	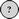	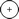	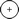	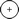	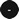
24. MacLean *et al*. (1989)	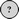	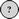	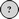	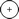	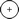	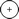	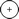
25. McKay *et al*. (1996*a*)	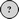	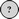	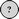	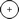	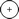	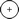	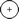
26. McKay *et al*. (1996*b*)	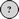	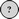	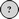	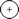	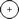	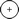	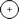
27. McKay *et al*. (1998)	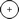	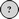	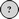	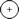	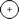	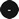	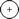
28. Parrish *et al*. (1986)	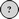	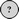	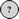	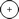	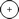	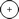	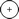
29. Planos and Glenwich (1986)	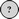	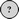	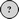	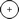	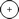	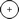	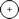
30. Richardson *et al*. (2014)	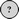	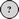	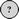	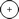	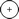	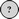	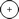
31. Stern *et al*. (2015)	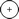	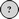	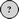	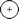	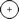	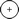	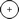
32. Stevens *et al*. (2009)	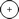	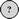	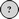	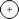	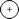	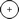	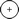
33. Szapocznik *et al*. (1988)	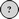	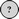	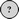	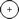	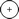	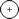	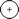
34. Wiseman and McBride (1998)	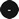	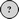	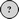	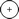	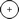	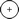	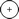

a. Random sequence generation; b. Allocation concealment; c. Blinding of participants and personnel; d. Blinding of outcome assessment; e. Incomplete outcome data; f. Selective reporting; g. Other bias; 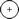 Low risk of bias; 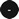 High risk of bias; 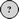 Unclear risk of bias.

*Reasons for assessment of high risk of bias: Husky *et al*. (2011): Consent obtained after randomisation; Jorm *et al*. (2010): Some schools switched into another group and randomisation of schools did not occur after baseline assessment; Lieberman *et al*. (2006): procedure for outcome assessment was different for intervention and control groups.

Although we did not formally grade the quality of evidence using the GRADE approach (Guyatt *et al*., 2011), we considered the criteria heterogeneity, risk of bias and precision where appropriate when reporting the effects of interventions below.

### Effects of interventions

**Fig. 3.**
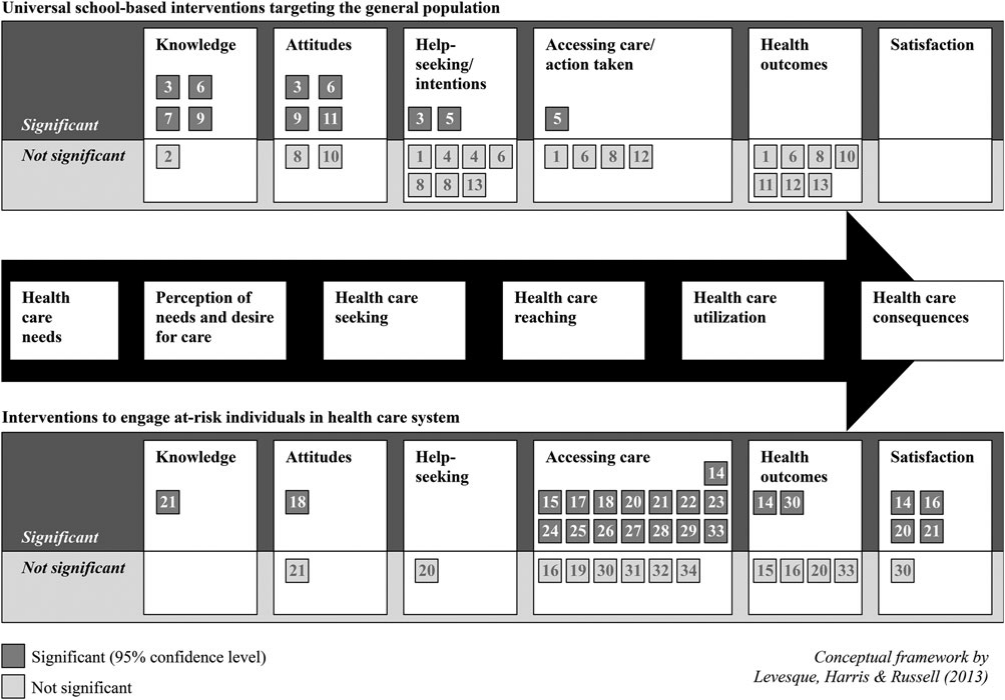
Significance of interventions' effect on targeted outcomes.

Figure 3 provides a graphical overview of which outcomes were measured by which studies and whether or not the interventions had a significant effect on the outcome measures. The full report of intervention effects can be found in Table 2, and details on how outcomes were defined in each study can be found in Appendix 6.

**Table 2.** Summary of findings

Universal school-based interventions targeting the general population									
	Author(s) (year)	Intervention description	Outcomes studied and effect sizes*						
			Knowledge	Attitudes	Intensions	Help-seeking	Action taken	Accessing care	Health outcomes
1	Aseltine *et al*. (2007)	Two-part suicide prevention program: (1) curriculum via video with dramatisations, interviews and guidelines including discussion guide over half of the school year; (2) screening on depression and suicidality symptoms				OR: 0.96 (0.82–1.12)		OR: 0.99 (0.78–1.25)	OR: 0.84 (0.69–1.02)
2	Campos *et al*. (2018)	Interactive two-session educational curriculum that explored students' knowledge and beliefs about physical and mental health and illness, identified risk factors and symptoms of mental disorders as well as help-seeking options, promoted non-stigmatised behaviours and inadequate beliefs related to mental disorders and addressed self-help strategies and mental health-promoting behaviour	ES: −0.06 (−0.26–0.14)						
3	Hart *et al*. (2018)	Three standardised pyschoeducation sessions via program booklet, presentations, videos, role-plays, group and workbook activities and final certificate for peer training to help adolescents with a mental health problem	**ES: 0.35 (0.23–0.47)**	**ES: 0.27 (0.15–0.38)**		**ES: 0.68 (0.56–0.80)**			
4	Howard *et al*. (2018)	Two brief online educational interventions consisting of one page of a vignette about a person with depression: a biological condition describing the biological causes of depression and a psychosocial condition describing the psychosocial causes of depression			Biological intervention ES: 0.25 (−0.26–0.76) Psychosocial intervention ES: 0.30 (−0.26–0.85)				
5	Husky *et al*. (2011)	Brief, two-stage mental health screening via questionnaire and structured interview; referrals to mental health care were provided for those who screened positive				**OR: 21.64 (6.66–70.36)**		**OR: 11.34 (3.41–37.69)**	
6	Jorm *et al*. (2010)	Two day-long teacher training sessions on common mental health disorders, school policy on mental health issues and how to assist students in need	**ES: 0.36 (0.14–0.58)**^a^	**OR: 2.56 (1.44–4.57)**^a^		OR: 1.27 (0.89–1.79)^b^	OR: 1.30 (0.82–2.08)^a^		OR: 1.08 (0.78–1.51)
7	Klingman and Hochdorf (1993)	Twelve weekly sessions delivering an educational curriculum on mental distress and disorders, help-seeking and prevention via theoretical component, role-playing and rehearsing new skills	**ES: 0.96 (0.48–1.43)**						
8	Morgan *et al*. (2019)	A 14-h standardised pyschoeducation and training program for parents of adolescents to learn to recognise early signs of a mental health problem or crisis and to assist adolescents to access appropriate professional help as early as possible		ES: 0.23 (−0.06–0.53)^b^	ES: −0.15 (−0.48–0.18)^b^	OR: 4.52 (0.57–35.75)	ES: 0.16 (−0.41–0.73)^b^		OR: 0.62 (0.16–2.34)
9	Painter *et al*. (2017)	Three-hour educational curriculum on stigma, mental illness and barriers to help-seeking via presentation, class discussion and video and presentation and discussion with a mentally ill college student	**ES: 0.31 (0.09–0.53)**	**ES: 0.42 (0.20–0.65)**					
10	Perry *et al*. (2014)	10 h of educational curriculum delivered over 5–8 weeks; resources for teachers delivering intervention included a booklet, slideshow and appendices on mental health, mood disorders and on helping others and oneself		ES: −0.02 (−0.29–0.26)					ES: −0.07 (−0.35–0.20)
11	Saporito *et al*. (2011)	A 35-min presentation on mental illness and its treatment plus video on an adolescent with a mental illness		**ES: 0.71 (0.39–1.04)**					ES: −0.31 (−0.63–0.00)
12	Sayal *et al*. (2010)	School-based screening to identify children with high ADHD rating scale scores and a book about ADHD and how to manage and work with affected children						OR: 1.22 (0.61–2.46)	OR: 1.12 (0.60–2.08)
13	Sharpe *et al*. (2017)	Booklets on mental health and disorders and help-seeking for students				ES: −0.02 (−0.01–0.05)^c^			ES: 0.01 (−0.03–0.04)^c^

a Teacher.

b Parent.

c Estimate for older age group (12–13 years).

**Table:** 

Interventions to engage at-risk individuals in health-care system								
	Author(s) (year)	Intervention description	Outcomes studied and effect sizes*					
			Knowledge	Attitudes	Help-seeking	Accessing care	Health outcomes	Satisfaction
14	Asarnow *et al*. (2005)	6-month health service quality improvement intervention through support and training for clinicians on treatment for people with mental disorders by expert leader teams and care managers				**OR: 2.17 (1.31–3.62)**	**ES: −0.25 (−0.46–0.03)**	**ES: 0.32 (0.10–0.53)**^1^
15	Asarnow *et al*. (2011)	Brief family-based therapy session to increase motivation during emergency room visit by reframing the problem, educating families about the importance of treatment, obtaining commitment from youth, identifying triggers, and developing and practising a safety plan supplemented by care linkage telephone contacts within the first 48 h after discharge				**OR: 3.65 (1.38–9.65)**	OR: 0.88 (0.23–3.40)	
16	Coker *et al*. (2019)	A 5-min video on community mental health clinic and scheduled visit for a telehealth eligibility screening				OR: 0.80 (0.40–1.62)	ES: −0.01 (−0.24–0.23)	**ES: 0.40 (0.17–0.64)**
17	Donohue *et al*. (1998)	Telephone call by clinical psychology doctoral students to parents about treatment, intake session for parent and youth, motivational reminder calls, and incentives to participate in treatment				**OR: 5.67 (1.02–31.54)**		
18	Fristad *et al*. (2003)	Didactic and interactive multi-family psycho-education group program; parent sessions focused on providing social support, information and skills, while child sessions focused on feeling less alone, understanding symptoms and effects of treatment and building social skills		**ES: 1.30 (0.45–2.16)**		**OR: 18.00 (2.47–131.29)**	Not reported	
19	Gadomski *et al*. (2010)	Three hour-long communication skills training sessions for primary care clinicians to engage parents and children in diagnosis and treatment and address barriers to treatment with group discussions and 10-min practice visits				ES: −0.03 (−0.23–0.16)		
20	Grupp-Phelan *et al*. (2012)	Discussion with a study social worker about screening results, patient concerns and available resources; designed to target various barriers and increase motivation for help-seeking behaviour			OR: 2.33 (0.42–43.20)	**OR: 9.62 (1.38–67.25)**	ES: 0.73 (−0.10–1.56)	**OR: 48.0 (3.70–622.0)**
21	Gully *et al*. (2008)	Educational booklet for parents on expectations and perceived value of treatment reviewed together with nurses	**ES: 2.18 (1.49–2.88)**	ES: 0.06 (−0.49–0.61)		**ES: 1.46 (0.84–2.07)**		**ES: 0.90 (0.32–1.47)**
22	Kourany *et al*. (1990)	Reminder telephone call, letter describing what would happen on the first clinic visit or both the call and the letter				**OR: 3.56 (1.28–9.94)**		
23	Lieberman *et al*. (2006)	Provision of on-site mental health services (usual care was a referral to an off-site mental health provider)				**OR: 74.0 (8.94–612.84)**		
24	MacLean *et al*. (1989)	One of four experimental letters (systematic appointment reminders, change slips requesting if appointment time should be changed, warnings and change slips combined with warnings)				**OR: 3.64 (1.40–9.48)**		
25	McKay *et al*. (1996*a*)	Intensive 30-min telephone intervention with a social worker to engage caretaker in help-seeking process by identifying child's problem, framing caretaker actions as having potential to impact the situation and exploring barriers to help-seeking				**OR: 3.22 (1.44–7.19)**		
26	McKay *et al*. (1996*b*)	Telephone intake with therapists trained in specific engagement skills, i.e., informing clients about the process of obtaining mental health services, responding to concrete concerns or crisis situations and exploring potential barriers to obtaining services				**OR: 4.16 (1.32–13.12)**		
27	McKay *et al*. (1998)	Thirty-minute telephone and in-person engagement intervention by master's level clinicians to clarify the need for mental health care, increase the caretaker's investment in help-seeking, identify attitudes about and previous experiences with mental health care and over concrete barriers to accessing services				**OR: 8.77 (3.41–22.54)**		
28	Parrish *et al*. (1986)	Letter informing parents that children would be moved to the bottom of the waiting list if three appointments were missed or letter informing parents that attending appointments would earn a coupon for winning a prize				**OR: 3.36 (1.40–8.03)**		
29	Planos and Glenwich (1986)	Appointment reminder (telephone or letter prompt)				**OR: 3.06 (1.33–7.05)**		
30	Richardson *et al*. (2014)	A 12-month collaborative care intervention delivered by master's-level clinicians involving initial in-person education engagement session, choice of treatment and regular follow-up				OR: 1.03 (0.42–2.51)	**ES: −0.57 (−1.02– −0.12)**	OR: 2.1 (0.7–6.1)
31	Stern *et al*. (2015)	A 10–15 min enhanced engagement phone intake to develop rapport with parents, identify and address likely barriers to treatment, increase parental self-efficacy, hope and treatment motivation				OR: 2.30 (0.97–5.46)		
32	Stevens *et al*. (2009)	Three phone calls in the first weeks after the first visit to the adolescent management clinic to assess youth's understanding of recommendations, address youth's struggles through case management and use motivational interviewing techniques if youth was ambivalent about treatment				OR: 1.10 (0.51–2.38)		
33	Szapocznik *et al*. (1988)	Engagement intervention during intake interview to overcome family's resistance to treatment by identifying family patterns that interfere with entry into treatment				**OR: 17.73 (5.58–56.34)**	ES: −0.62 (−1.01– −0.24)	
34	Wiseman and McBride (1998)	A letter stating that confirmation from parents was required if they still wanted an appointment				OR: 2.30 (0.94–5.61)		

ES, effect size; OR, odds ratio.

* Significant/not significant at 95% confidence level.

Among the studies on universal school-based interventions targeting the general population, 80% (4/5) of those that assessed knowledge about accessing mental health care, 67% (4/6) of those that assessed attitudes or beliefs about seeking care, 22% (2/9) of those that assessed help-seeking or intentions, 20% (1/5) of those that assessed accessing care or taking action and none (0/7) of those that assessed mental health outcomes had a significant impact on the respective outcome. Thus, universal school-based interventions targeting individuals from the general population tended to have a significant impact on steps earlier on the pathway to accessing care, especially knowledge and attitudes, but not on later steps, such as actually accessing care or mental health outcomes. The risk of bias for studies on these interventions ranged from low to high (see Table 1). The effect sizes ranged from −0.06 to 0.96 for knowledge about seeking health care, −0.02 to 2.56 for attitudes about seeking health care and −0.15 to 0.30 for intensions to seek health care or help others seek health care. Both odds ratios and effect sizes were calculated for the outcomes help-seeking, action taken and health outcomes. The odds ratios ranged from 0.96 to 21.64 for help-seeking, 0.99 to 11.34 for accessing care and 0.62 to 1.12 for health outcomes.

The pattern of significant outcomes found for universal interventions differed from that observed for interventions targeting at-risk individuals who had already been identified by the health-care system. Among studies on these interventions, all assessed accessing care (e.g., the proportion of study subjects who attended the first appointment or number of appointments attended) as an outcome, and 71% (15/21) of these interventions had a significant impact on that outcome. Eighty per cent (8/10) of studies on treatment engagement interventions (e.g., a family-based session to increase motivation during an emergency room or motivational telephone calls with trained staff) and 80% (4/5) of studies on appointment reminder interventions had a significant effect on accessing care. Just three interventions targeting at-risk individuals assessed outcomes that preceded accessing care. The effects on knowledge about accessing mental health care, attitudes or beliefs about seeking care and help-seeking were thus unclear due to the limited number of studies measuring these outcomes. Of the seven interventions that assessed the remaining two outcomes along the pathway to accessing care, 33% (2/6) of those that assessed mental health outcomes and 80% (4/5) of those that assessed satisfaction with care were significantly better as compared with controls on the respective outcome. Interventions targeting at-risk children who had already been identified by the health-care system therefore generally yielded more access to care and satisfaction with care as compared with controls, but not necessarily improved mental health outcomes. The risk of bias found for appointment reminder and treatment engagement interventions ranged from low to high (see Table 1). The most important outcome comparisons for these types of interventions are summarised in the meta-analyses below.

### Meta-analyses

**Fig. 4.**
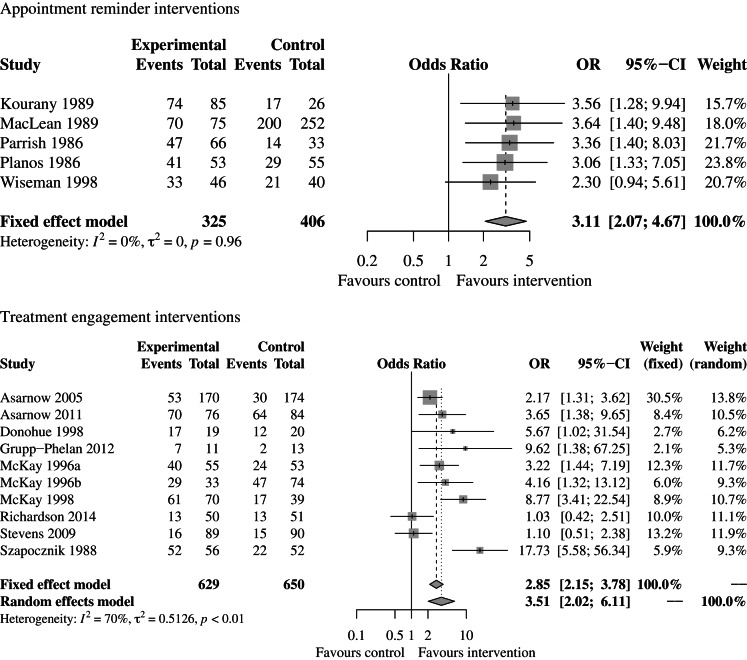
Forest plots of appointment reminder and treatment engagement interventions measuring first appointment attendance.

We conducted meta-analyses for two types of interventions that measured the same outcome (accessing care) using the binary measure first appointment attendance (yes/no): (1) appointment reminder interventions (five studies) and (2) treatment engagement interventions (10 studies). For the appointment reminder interventions, we only calculated a fixed-effects model since heterogeneity was low (*I*^2^ = 0%). For the treatment engagement interventions, heterogeneity was substantial (*I*^2^ = 70%), so we calculated fixed effects and random-effects models. Forest plots for each of these two types of interventions can be found in Fig. 4. The combined odds ratio of the appointment reminder interventions was 3.11 (2.07–4.67), and the combined odds ratio calculated using the random-effects model for the treatment engagement interventions was 3.51 (2.02–6.11). In other words, the odds of attending an initial appointment were 3.11 times higher for those who received an appointment reminder as compared with controls and 3.51 higher for those who participated in a treatment engagement intervention as compared with controls, indicating that overall, both types of interventions yielded significantly higher first appointment attendance in the target population as compared with controls.

## Discussion

### Summary of main results

This systematic review identified 34 RCTs of interventions that fell into two main categories: universal school-based interventions targeting the general population and interventions to engage at-risk individuals who had already been identified by the health-care system. Interventions in the first category generally yielded significantly better knowledge and attitudes about accessing care as compared with controls, but did not have an impact on actually accessing care or on mental health outcomes. Most interventions targeting at-risk children who had already been identified by the health-care system yielded significantly better access to care and satisfaction with care as compared with controls, but did not seem to have a significant impact on mental health outcomes. Meta-analyses of appointment reminder interventions and treatment engagement interventions measuring the outcome accessing care using the binary measure first appointment attendance found that both types of interventions yielded significantly more access to care as compared with controls. We did not identify studies that targeted the domains of acceptability and affordability or individuals' ability to reach or pay for care.

### Comparison with other reviews

In our study, we used Levesque *et al*.'s conceptual framework of access to health care to design a systematic review of RCTs of interventions to improve access to mental health care for children (Levesque *et al*., 2013). This approach enabled us to provide a broad overview of RCTs of interventions that was not limited to particular types of interventions or disorders by structuring our findings along the entire pathway to accessing care. Our results on the effects of universal interventions targeting individuals from the general population were similar to those from a previous systematic review on help-seeking interventions for depression, anxiety and general psychological distress in adults that found that mental health literacy content significantly improved help-seeking attitudes, but did not have an effect on help-seeking behaviour (Gulliver *et al*., 2012). Another systematic review on interventions to promote help-seeking for mental health problems found that interventions increased formal help-seeking behaviours when targeting affected or at-risk people with mental disorders, but not the general population (Xu *et al*., 2018). This is the same pattern that we found for the outcome of accessing care.

### Strengths and limitations

This systematic review provided an overview of interventions to improve access to mental health care along the entire pathway to accessing care using a conceptual framework, which allowed us to assess where evidence for effective interventions lies and where evidence is missing. However, taking a broad approach to the search necessitated restricting the search to the title field and English language only. We attempted to address this by hand searching reference lists and key articles. In addition, we did not include retention in treatment as an outcome in this review since we were interested in gaining access to treatment.

### Implications for practice and research

Both the results of this systematic review and previous research have shown that interventions can improve knowledge and attitudes about mental disorders and their treatment (Lo *et al*., 2017); however, there is evidence that such interventions do not necessarily have an impact on health behaviours, such as help-seeking (Kelly and Barker, 2016; Laverack, 2017). Interventions to engage and motivate an at-risk population, on the other hand, have been shown to significantly change health behaviours (Ingoldsby, 2010). From a public health perspective, the problem with this finding is that existing interventions do not improve access to care for people in need from the general population, leaving a large treatment gap.

In order to have a population-level effect on improving access to care, it may be necessary to introduce two-stage interventions, i.e., ones that first identify those in need from the general population and then engage them in the health-care system. In this systematic review, the only study in that targeted the general population, yet had a significant impact on accessing care was Husky *et al*. (2011), which tested systematic referral to mental health services using a brief mental health screening in a school setting (Husky *et al*., 2011). Additionally, five of the six studies targeting at-risk children that took place in a primary care setting used a screening procedure to identify these at-risk children; however, only two of these five studies had a significant effect on access to care as compared with controls. Since the evidence to recommend screening the general population of children for mental disorders is currently insufficient (Lenzer, 2017), it is imperative to rigorously test screening procedures using RCTs giving careful consideration to the benefits and harms that would result from such screenings (Wissow *et al*., 2013).

There is growing evidence that changing environmental factors, including policies, infrastructure and health-care financing (Hodgkinson *et al*., 2017; So *et al*., 2019), can have a larger impact on health behaviours such as help-seeking than health literacy education (Kelly and Barker, 2016). Integrating mental health services into existing service settings is considered a promising means of improving access to care (Anderson *et al*., 2017; Hodgkinson *et al*., 2017; Richardson *et al*., 2017); however, we identified just one infrastructural intervention that involved providing onsite mental health services for primary care patients (Lieberman *et al*., 2006). In light of this and the fact that our systematic review revealed gaps in the research on interventions to improve acceptability, affordability and individuals' ability to reach and pay, it seems that more research on interventions that address contextual factors such as these is warranted, although it may be difficult to test some of these interventions via RCT. In addition, targeting individual barriers in isolation, such as cost or insurance coverage, without addressing other barriers like accessibility, acceptability and availability may not improve service utilisation (So *et al*., 2019). It is possible that interventions that address multiple barriers simultaneously are more likely to have a population-level effect on improving children's access to mental health care, but this must be tested in future research.

Future studies on interventions to improve access to mental health care for young people should attempt to coordinate and standardise the outcomes assessed so that more quantitative comparison among studies via meta-analysis is possible. We especially need more studies testing the effects on mental health care outcomes since this is the ultimate purpose of improving access to care. In addition, longer follow-up periods are required to gain information about the longer-term effects of interventions to improve access to care (Salerno, 2016; Anderson *et al*., 2017). Finally, none of the studies identified in this systematic review took place in low- or middle-income countries. Due to a shortage of mental health professionals, the fact that detection rates of mental disorders are much lower in many of these countries, less developed infrastructure and potentially more stigma surrounding mental health disorders, different interventions than those that are effective in high-income countries may be required (Patel *et al*., 2013). More research is therefore needed to draw conclusions about improving access to care in these settings.

## Conclusion

In order to bridge the existing treatment gap in mental health care for children, interventions that aim to improve knowledge and attitudes about mental health care in the general population are not sufficient. Instead, a two-stage approach that first identifies young people in need of care from the general population and then engages them in the health-care system should be tested in high quality studies. In addition, we need high quality research on the impact of interventions addressing contextual factors such as affordability and individuals' ability to reach care.

## Data

The protocol for this systematic review has been registered with the International Prospective Register of Systematic Reviews (PROSPERO, ID: CRD42018081714). The publication details for the studies included in this systematic review have been included in the reference list.

## Appendix 1

A conceptual framework of access to health care (Levesque *et al*., 2013)

## Appendix 2

PRISMA checklist (Moher *et al*., 2009)

**Table:**

Section/topic	#	Checklist item	Reported on page #
*Title*			
Title	1	Identify the report as a systematic review, meta-analysis or both.	1
*Abstract*			
Structured summary	2	Provide a structured summary including as applicable: background; objectives; data sources; study eligibility criteria, participants and interventions; study appraisal and synthesis methods; results; limitations; conclusions and implications of key findings; systematic review registration number.	1
*Introduction*			
Rationale	3	Describe the rationale for the review in the context of what is already known.	1–2
Objectives	4	Provide an explicit statement of questions being addressed with reference to participants, interventions, comparisons, outcomes and study design (PICOS).	2
*Methods*			
Protocol and registration	5	Indicate if a review protocol exists, if and where it can be accessed (e.g., Web address) and, if available, provide registration information including registration number.	2
Eligibility criteria	6	Specify study characteristics (e.g., PICOS, length of follow-up) and report characteristics (e.g., years considered, language, publication status) used as criteria for eligibility, giving rationale.	2
Information sources	7	Describe all information sources (e.g., databases with dates of coverage, contact with study authors to identify additional studies) in the search and date last searched.	2
Search	8	Present full electronic search strategy for at least one database, including any limits used, such that it could be repeated.	Appendix 3
Study selection	9	State the process for selecting studies (i.e., screening, eligibility, included in systematic review and, if applicable, included in the meta-analysis).	2–3
Data collection process	10	Describe the method of data extraction from reports (e.g., piloted forms, independently, in duplicate) and any processes for obtaining and confirming data from investigators.	2–3
Data items	11	List and define all variables for which data were sought (e.g., PICOS, funding sources) and any assumptions and simplifications made.	2
Risk of bias in individual studies	12	Describe methods used for assessing risk of bias of individual studies (including specification of whether this was done at the study or outcome level), and how this information is to be used in any data synthesis.	3, 5
Summary measures	13	State the principal summary measures (e.g., risk ratio, difference in means).	3–4
Synthesis of results	14	Describe the methods of handling data and combining results of studies, if done, including measures of consistency (e.g., *I*^2^) for each meta-analysis.	3–4
Risk of bias across studies	15	Specify any assessment of risk of bias that may affect the cumulative evidence (e.g., publication bias, selective reporting within studies).	N/A
Additional analyses	16	Describe methods of additional analyses (e.g., sensitivity or subgroup analyses, meta-regression), if done, indicating which were pre-specified.	3–4
*Results*			
Study selection	17	Give numbers of studies screened, assessed for eligibility and included in the review, with reasons for exclusions at each stage, ideally with a flow diagram.	4–5, Fig. 1
Study characteristics	18	For each study, present characteristics for which data were extracted (e.g., study size, PICOS, follow-up period) and provide the citations.	4–5, Table 2, Appendix 5
Risk of bias within studies	19	Present data on risk of bias of each study and, if available, any outcome level assessment (see item 12).	5, Table 1
Results of individual studies	20	For all outcomes considered (benefits or harms), present, for each study: (a) simple summary data for each intervention group (b) effect estimates and confidence intervals, ideally with a forest plot.	Table 2, Fig. 4
Synthesis of results	21	Present results of each meta-analysis done, including confidence intervals and measures of consistency.	7
Risk of bias across studies	22	Present results of any assessment of risk of bias across studies (see Item 15).	N/A
Additional analysis	23	Give results of additional analyses, if done (e.g., sensitivity or subgroup analyses, meta-regression [see Item 16]).	Figures 2 and 3
*Discussion*			
Summary of evidence	24	Summarise the main findings including the strength of evidence for each main outcome; consider their relevance to key groups (e.g., healthcare providers, users and policymakers).	7–12
Limitations	25	Discuss limitations at study and outcome level (e.g., risk of bias), and at review-level (e.g., incomplete retrieval of identified research, reporting bias).	8
Conclusions	26	Provide a general interpretation of the results in the context of other evidence and implications for future research.	7–12
*Funding*			
Funding	27	Describe sources of funding for the systematic review and other support (e.g., supply of data); role of funders for the systematic review.	N/A

## Appendix 3

Search strategy

**Table:**

Search in EMBASE Date of search: 15 May 2019		
No.	Query	Results
# 1	‘mental disease'/exp OR 'mental health’/exp OR (((mental OR mentally OR psychiatric OR psychological OR psychosocial OR behavioural OR behavioural OR emotional) NEAR/3 (health OR disease OR diseases OR disorder OR disorders OR ill OR illness OR illnesses OR insanity OR insanities OR abnormality OR abnormalities OR disturbance OR disturbances OR confusion OR confusions OR symptom OR symptoms OR health OR problem OR problems)):ti) OR depression:ti OR depressive:ti	2269428
# 2	‘adolescent’/exp OR ‘child’/exp OR child:ti OR children:ti OR adolescent:ti OR adolescents:ti OR juvenile:ti OR juveniles:ti OR young:ti OR youth:ti OR pediatric*:ti OR paediatric*:ti OR teen*:ti OR ‘young people’:ti OR ‘young person*’:ti OR minor*:ti	3820655
# 3	‘help seeking behavior’/exp OR (((help OR treatment OR treatments) NEAR/5 (seek* OR behavior OR behaviour)):ti) OR helpseeking:ti OR ‘mental health literacy’:ti OR (((screening* OR intervention* OR communica* OR utili?ation OR access OR attitude*) NEAR/5 (mental OR psych*)):ti)	31782
# 4	#1 AND #2 AND #3	4583
# 5	#1 AND #2 AND #3 NOT ([animals]/lim NOT [humans]/lim) AND [english]/lim NOT [conference abstract]/lim	3676

Note: This search strategy was adapted to search MEDLINE, PsycINFO and the Cochrane Central Register of Controlled Trials (CENTRAL)

## Appendix 4

List of studies excluded from the review by primary reason for exclusion

Reason 1. Not an RCT (*n* = 16)

1. **Battaglia J, Coverdale JH and Bushong CP** (1990) Evaluation of a Mental Illness Awareness Week program in public schools. *American Journal of Psychiatry* 147, 324.
2. **Contreras S, Porras-Javier L, Zima BT, Soares N, Park C, Patel A, Chung PJ and Coker TR** (2018) Development of a telehealth-coordinated intervention to improve access to community-based mental health. *Ethnicity & Disease* 28(Supp), 457–466.
3. **Cynthia Logsdon M, Myers J, Rushton J, Gregg JL, Josephson AM, Davis DW, Brothers K, Baisch K, Carabello A, Vogt K, Jones K and Angermeier J** (2018) Efficacy of an Internet-based depression intervention to improve rates of treatment in adolescent mothers. *Archives of Women*'*s Mental Health* 21, 273–285.
4. **Elliott DJ, Koroloff NM, Koren PE and Friesen BJ** (1998) Improving access to children's mental health services: the Family Associate approach. In Epstein MH *et al*. (eds), *Outcomes for Children and Youth with Emotional and Behavioral Disorders and their Families: Programs and Evaluation Best Practices.* pp. 581–609.
5. **Esters IG, Cooker PG and Ittenbach RF** (1998) Effects of a unit of instruction in mental health on rural adolescents' conceptions of mental illness and attitudes about seeking help. *Adolescence* 33, 469–476.
6. **Grimes KE, Creedon TB, Webster CR, Coffey SM, Hagan GN and Chow CM** (2018) Enhanced child psychiatry access and engagement via integrated care: A collaborative practice model with pediatrics. *Psychiatric Services* 69, 986–992.
7. **Jonovich SJ and Alpert-Gillis LJ** (2014) Impact of pediatric mental health screening on clinical discussion and referral for services. *Clinical Pediatrics* 53, 364–371.
8. **Lubman DI, Cheetham A, Berridge BJ and McKay-Brown L** (2018) MAKINGtheLINK: A school-based intervention to improve help-seeking for substance use problems. *Early Intervention in Psychiatry* 12, 915–921.
9. **McKay MM, Hibbert R, Hoagwood K, Rodriguez J, Murray L, Legerski J and Fernandez D** (2004) Integrating evidence-based engagement interventions into ‘real world' child mental health settings. *Brief Treatment and Crisis Intervention* 4, 177–186.
10. **Rotheram-Borus MJ, Piacentini J, Van Rossem R, Graae F, Cantwell C, Castro-Blanco D, Miller S and Feldman J** (1996) Enhancing treatment adherence with a specialized emergency room program for adolescent suicide attempters. *Journal of the American Academy of Child and Adolescent Psychiatry* 35, 654–663.
11. **Ruble AE, Leon PJ, Gilley-Hensley L, Hess SG and Swartz KL** (2013) Depression knowledge in high school students: Effectiveness of the adolescent depression awareness program. *Journal of Affective Disorders* 150, 1025–1030.
12. **Shaffer D, Garland ANN, Vieland V, Underwood M and Busner C** (1991) The impact of curriculum-based suicide prevention programs for teenagers. *Journal of the American Academy of Child & Adolescent Psychiatry* 30, 588–596.
13. **Smalec JL and Klingle RS** (2000) Bulimia interventions via interpersonal influence: The role of threat and efficacy in persuading bulimics to seek help. *Journal of Behavioral Medicine* 23, 37–57.
14. **Spirito A, Overholser J, Ashworth S, Morgan J and Benedict-Drew C** (1988) Evaluation of a suicide awareness curriculum for high school students. *Journal of the American Academy of Child & Adolescent Psychiatry* 27, 705–711.
15. **Swartz KL, Kastelic EA, Hess SG, Cox TS, Gonzales LC, Mink SP and Raymond DePaulo J** (2010) The effectiveness of a school-based adolescent depression education program. *Health Education & Behavior* 37, 11–22.
16. **Ventieri D, Clarke DM and Hay M** (2011) The effects of a school-based educational intervention on pre-adolescents' knowledge of and attitudes towards mental illness. *Advances in School Mental Health Promotion* 4, 5–17.

Reason 2. Trial not yet completed (*n* = 4)

1. **Bauer S, Bilić S, Reetz C, Ozer F, Becker K, Eschenbeck H, Kaess M, Rummel-Kluge C, Salize HJ, Diestelkamp S and Moessner M** (2019) Efficacy and cost-effectiveness of Internet-based selective eating disorder prevention: study protocol for a randomized controlled trial within the ProHEAD Consortium. *Trials* 20.
2. **Calear AL, Banfield M, Batterham PJ, Morse AR, Forbes O, Carron-Arthur B and Fisk M** (2017) Silence is deadly: a cluster-randomised controlled trial of a mental health help-seeking intervention for young men. *BMC Public Health* 17, 834.
3. **Darraj H, Mahfouz MS, Al Sanosi R, Badedi M and Sabai A** (2018) The effects of an educational program on depression literacy and stigma among students of secondary schools in Jazan city, 2016. *Medicine* 97, e9433.
4. **Kilbourne AM, Smith SN, Choi SY, Koschmann E, Liebrecht C, Rusch A, Abelson JL, Eisenberg D, Himle JA, Fitzgerald K and Almirall D** (2018) Adaptive School-based Implementation of CBT (ASIC): clustered-SMART for building an optimized adaptive implementation intervention to improve uptake of mental health interventions in schools. *Implementation Science* 13, 119.

Reason 3. Did not target population of interest (*n* = 2)

1. **Weinstein M** (1988) Preparation of children for psychotherapy through videotaped modeling. *Journal of Clinical Child Psychology* 17, 131–136.
2. **Winkler P, Janoušková M, Kožený J, Pasz J, Mladá K, Weissová A, Tušková E and Evans-Lacko S** (2017) Short video interventions to reduce mental health stigma: a multi-centre randomised controlled trial in nursing high schools. *Social Psychiatry and Psychiatric Epidemiology* 52, 1549–1557.

Reason 4. Did not include outcome of interest (*n* = 10)*

1. **Baer JS, Garrett SB, Beadnell B, Wells EA and Peterson PL** (2007) Brief motivational intervention with homeless adolescents: evaluating effects on substance use and service utilization. *Psychology of Addictive Behaviors* 21, 582–586.
2. **Burns BJ, Farmer EMZ, Angold A, Costello EJ and Behar L** (1996) A randomized trial of case management for youths with serious emotional disturbance. *Journal of Clincial Child Psychology* 25, 376–387.
3. **Fristad MA** (2006) Psychoeducational treatment for school-aged children with bipolar disorder. *Development and Psychopathology* 18.
4. **Nock MK and Kazdin AE** (2005) Randomized controlled trial of a brief intervention for increasing participation in parent management training. *Journal of Consulting and Clinical Psychology* 73, 872–879.
5. **Pinto-Foltz MD, Logsdon MC and Myers JA** (2011) Feasibility, acceptability, and initial efficacy of a knowledge-contact program to reduce mental illness stigma and improve mental health literacy in adolescents. *Social Science and Medicine* 72, 2011–2019.
6. **Rahman A, Mubbashar MH, Gater R and Goldberg D** (1998) Randomised trial of impact of school mental-health programme in rural Rawalpindi, Pakistan. *Lancet* 352, 1022–1025.
7. **Sakellari E, Sourander A, Kalokerinou-Anagnostopoulou A and Leino-Kilpi H** (2016) Opinions about mental illness among adolescents: the impact of a mental health educational intervention. *School Mental Health* 8, 268–277.
8. **Vila-Badia R, Martínez-Zambrano F, Arenas O, Casas-Anguera E, García-Morales E, Villellas R, Martín JR, Pérez-Franco MB, Valduciel T, Casellas D, García-Franco M, Miguel J, Balsera J, Pascual G, Julia E and Ochoa S** (2016) Effectiveness of an intervention for reducing social stigma towards mental illness in adolescents. *World Journal of Psychiatry* 6, 239–247.
9. **Wagner V, Sy J, Weeden K, Blanchard T, Cauce AM, Morgan CJ, Moore E, Wurzbacher K and Tomlin S** (1994) Effectiveness of intensive case management for homeless adolescents: results of a 3-month follow-up. *Journal of Emotional and Behavioral Disorders* 2, 219–227.
10. **Wissow LS, Gadomski A, Roter D, Larson S, Brown J, Zachary C, Bartlett E, Horn I, Luo X and Wang M-C** (2008) Improving child and parent mental health in primary care: a cluster-randomized trial of communication skills training. *Pediatrics* 121, 266–275.

Reason 5. Control group not appropriate (*n* = 1)

1. **Chisholm K, Patterson P, Torgerson C, Turner E, Jenkinson D and Birchwood M** (2016) Impact of contact on adolescents' mental health literacy and stigma: the SchoolSpace cluster randomised controlled trial. *BMJ Open* 6, e009435.

Reason 6. Not possible to calculate effect size (*n* = 4)

1. **Beaudry MB, Swartz K, Miller L, Schweizer B, Glazer K and Wilcox H** (2019) Effectiveness of the Adolescent Depression Awareness Program (ADAP) on depression literacy and mental health treatment. *Journal of School Health* 89, 165–172.
2. **Poland AL** (2010) *Got Training? The Effect of Mental Health Training on the Attitudes and Behaviors of Direct Care Workers in a Residential Facility for Juvenile Offenders*. Ipswich, MA: ProQuest Information & Learning.
3. **Warzak WJ, Parrish JM and Handen BL** (1987) Effects of telephone intake procedures on initial appointment keeping in a child behavior management clinic. *Journal of Compliance in Health Care* 2, 143–154.
4. **Watt BD, Hoyland M, Best D and Dadds MR** (2007) Treatment participation among children with conduct problems and the role of telephone reminders. *Journal of Child and Family Studies* 16, 522–530.

*For a study with outcomes on health measures and satisfaction with care to be included in the analysis, the study also had to include a measure about improved access to care.

## Appendix 5

Characteristics of included studies by intervention type

**Table:**

Universal school-based interventions targeting the general population										
No.	First author, year/country	*N* enrolled/analysed	Age	Setting	Targeted population	Target condition	Intervention description	Intervention duration	Assessment time point(s)	Outcomes studied
1	Aseltine, 2007/USA	4491/3837–3899	14–18	School	Students	Suicide	Two-part suicide prevention program: (1) curriculum via video with dramatisations, interviews and guidelines including discussion guide over half of the school year; (2) screening on depression and suicidality symptoms	2 days	3 months	– Help-seeking – Accessing care – Health outcomes
2	Campos, 2018/Portugal	543/387	12–14	School	Students	General mental health problems	Interactive two-session educational curriculum that explored students' knowledge and beliefs about physical and mental health and illness, identified risk factors and symptoms of mental disorders as well as help-seeking options, promoted non-stigmatised behaviours and inadequate beliefs related to mental disorders and addressed self-help strategies and mental health-promoting behaviour	Two 90-min sessions (1 week apart)	– 1 week (post-intervention) – 6 months	Knowledge
3	Hart, 2018/Australia	1942/1116	15–18	School	Student peers, parents and teachers	General mental health problems	Three standardised pyschoeducation sessions via program booklet, presentations, videos, role-plays, group and workbook activities and final certificate for peer training to help adolescents with a mental health problem	3 weeks	1 week	– Knowledge – Attitudes – Intentions
4	Howard, 2018/Australia	350/327	16–19	School	Students	Depression	Two brief online educational interventions consisting of one page of a vignette about a person with depression: a biological condition describing the biological causes of depression and a psychosocial condition describing the psychosocial causes of depression	Single time point	Directly post-intervention	Intensions
5	Husky, 2011/USA	890/656	14–15	School	Students	General mental health problems	Brief, two-stage mental health screening via questionnaire and structured interview; referrals to mental health care were provided for those who screened positive	Single time point	3–5 months	– Help-seeking – Accessing care
6	Jorm, 2010/Australia	423/327	12–15	School	Teachers	General mental health problems	Two day-long teacher training sessions on common mental health disorders, school policy on mental health issues and how to assist students in need	Two days	– Directly post-intervention – 6 months	– Knowledge – Attitudes – Intentions – Action taken – Health outcomes
7	Klingman, 1993/Israel	237/76	12–13	School	Student peers	Mental distress and suicide	Twelve weekly sessions delivering an educational curriculum on mental distress and disorders, help-seeking and prevention via theoretical component, role-playing and rehearsing new skills	12 weeks	2 weeks	Knowledge
8	Morgan, 2019/Australia	384/322	12–15	School	Parents of adolescents	General mental health problems	A 14-h standardised pyschoeducation and training program for parents of adolescents to learn to recognise early signs of a mental health problem or crisis and to assist adolescents to access appropriate professional help as early as possible	Two sessions over four months	– 1 year – 2 years	– Attitudes – Intensions – Action taken – Help-seeking – Health outcomes
9	Painter, 2017/USA	751/721	10–13	School	Students	General mental health problems	Three-hour educational curriculum on stigma, mental illness and barriers to help-seeking via presentation, class discussion and video and presentation and discussion with a mentally ill college student	⩽1 week	1 week	– Knowledge – Attitudes
10	Perry, 2014/Australia	380/208	13–16	School	Students	General mental health problems	Ten hour of educational curriculum delivered over 5–8 weeks; resources for teachers delivering intervention included a booklet, slideshow and appendices on mental health, mood disorders and on helping others and oneself	5–8 weeks	– Post-intervention – 6 months	– Attitudes – Health outcomes
11	Saporito, 2011/USA	159/156	15–19	School	Students	General mental health problems	A35-min presentation on mental illness and its treatment plus video on an adolescent with a mental illness	Single time point	Directly post-intervention	– Attitudes – Health outcomes
12	Sayal, 2010/UK	1662/487	4–5	School	Students and teachers	Attention-deficit hyperactivity disorder	School-based screening to identify children with high ADHD rating scale scores and a book about ADHD and how to manage and work with affected children	Single time point	5 years	– Accessing care – Health outcomes
13	Sharpe, 2017/UK	27▫885/ 14▫690	10–13	School	Students	General mental health problems	Booklets on mental health and disorders and help-seeking for students	Single time point	12 months	– Help-seeking – Health outcomes

**Table:**

Interventions to engage at-risk individuals within health-care system										
No.	First author, year/country	*N* enrolled/analysed	Age	Setting	Targeted population	Target condition	Intervention description	Intervention duration	Assessment time point(s)	Outcomes studied
14	Asarnow, 2005/USA	418/344	13–21	Primary care	Patients with depressive symptoms and their parents (when appropriate)	Depression	6-month health service quality improvement intervention through support and training for clinicians on treatment for people with mental disorders by expert leader teams and care managers	6 months	6 months	– Accessing care – Health outcomes – Satisfaction
15	Asarnow, 2011/USA	181/160	10–18	Emergency department	Suicidal youths and their families	Suicide	Brief family-based therapy session to increase motivation during emergency room visit by reframing the problem, educating families about the importance of treatment, obtaining commitment from youth, identifying triggers and developing and practising a safety plan supplemented by care linkage telephone contacts within the first 48 h after discharge	Single time point with follow-up phone calls after 48 h and additional contacts as needed (usually 1, 2 and 4 weeks post-discharge)	2 months	– Accessing care – Health outcomes
16	Coker, 2019/USA	342/342	5–12	Primary care	Parents of children referred to community mental health clinics	General mental health problems	A 5-min video on community mental health clinic and scheduled visit for a telehealth eligibility screening	Single time point	6 months	– Accessing care – Health outcomes – Satisfaction
17	Donohue, 1998/USA	39/39	Not stated (ca. 12–18)	Outpatient cognitive-behavioural treatment program specialising in adolescent substance dependence and conduct disorder	Adolescents referred as prospective clients and their parents	Conduct disorder and substance abuse	Telephone call by clinical psychology doctoral students to parents about treatment, intake session for parent and youth, motivational reminder calls and incentives to participate in treatment	Single time point	Directly post-intervention	Accessing care
18	Fristad, 2003/USA	52/42	8–11	Clinical research group	Children with mood disorders and their parents	Mood disorders	Didactic and interactive multi-family psycho-education group program; parent sessions focused on providing social support, information and skills, while child sessions focused on feeling less alone, understanding symptoms and effects of treatment and building social skills	6 sessions over 6 weeks	– 2 months – 6 months	– Attitudes – Accessing care – Health outcomes
19	Gadomski, 2010/USA	397/397	5–16	Primary care	Primary care providers who treat children with possible or probable mental health problems	General mental health problems	Three hour-long communication skills training sessions for primary care clinicians to engage parents and children in diagnosis and treatment and address barriers to treatment with group discussions and 10-min practice visits	Single time point	– 2 weeks – 3 months – 6 months	Accessing care
20	Grupp-Phelan, 2012/USA	24/24	12–17	Emergency department	Patients with suicide-related risk factors	Suicide	Discussion with a study social worker about screening results, patient concerns and available resources; designed to target various barriers and increase motivation for help-seeking behaviour	Single time point	2 months	– Help-seeking – Accessing care – Health outcomes – Satisfaction
21	Gully, 2008/USA	87/51	2–17	Child advocacy centres and outpatient program at hospital	Parents of children who are suspected victims of abuse	General mental health problems	Educational booklet for parents on expectations and perceived value of treatment reviewed together with nurses	Single time point	1 month	– Knowledge – Attitudes – Accessing care – Satisfaction
22	Kourany, 1989/USA	111/111	2–17	Outpatient child psychiatry clinic	Parents of prospective clients	General mental health problems	Reminder telephone call, letter describing what would happen on the first clinic visit, or both the call and the letter	Single time point	Directly post-intervention	Accessing care
23	Lieberman, 2006/USA	71/71	13–22	Primary care	Adolescents with psychosocial issues	General mental health problems	Provision of on-site mental health services (usual care was a referral to an off-site mental health provider)	Single time point	3 months	Accessing care
24	MacLean, 1989/Canada	327/327	<12	Child community mental health centre	Parents of prospective clients	Non-emergency general mental health problems	One of four experimental letters (systematic appointment reminders, change slips requesting if appointment time should be changed, warnings and change slips combined with warnings)	Single time point	Directly post-intervention	Accessing care
25	McKay, 1996a/USA	108/108	Not stated	Child mental health agency	Caretakers requesting mental health services	General mental health problems	Intensive 30-min telephone intervention with a social worker to engage caretaker in help-seeking process by identifying child's problem, framing caretaker actions as having potential to impact the situation, and exploring barriers to help-seeking	Single time point	Directly post-intervention	Accessing care
26	McKay, 1996b/USA	107/107	Not stated	Urban child mental health agency	Parents of prospective clients	Non-emergency general mental health problems	Telephone intake with therapists trained in specific engagement skills, i.e., informing clients about the process of obtaining mental health services, responding to concrete concerns or crisis situations and exploring potential barriers to obtaining services	Single time point	Directly post-intervention	Accessing care
27	McKay, 1998/USA	109/109	1–14	Child mental health agency	Caregivers of urban children who requested services at the mental health agency	General mental health problems	Thirty-minute telephone and in-person engagement intervention by master's level clinicians to clarify the need for mental health care, increase the caretaker's investment in help-seeking, identify attitudes about and previous experiences with mental health care, and over concrete barriers to accessing services	Single time point	18 weeks	Accessing care
28	Parrish, 1986/USA	99/99	2–20	Outpatient behavioural paediatrics clinic	Parents of children referred as prospective clients	Behavioural health problems	Letter informing parents that children would be moved to the bottom of the waiting list if three appointments were missed or letter informing parents that attending appointments would earn a coupon for winning a prize	Single time point	Directly post-intervention	Accessing care
29	Planos, 1986/USA	274/274	<18	Children's mental health centre	Parents of children referred as prospective clients	General mental health problems	Appointment reminder (telephone or letter prompt)	Single time point	1 month	Accessing care
30	Richardson, 2014/USA	101/101	13–17	Primary care	Adolescents who screened positive for depression and their parents	Depression	A 12-month collaborative care intervention delivered by master's-level clinicians involving initial in-person education engagement session, choice of treatment and regular follow-up	12 months	12 months	– Accessing care – Health outcomes – Satisfaction
31	Stern, 2015/Canada	117/99	5–12	Children's mental health centre	Parents of children with mental health problems	General mental health problems	A 10–15 min enhanced engagement phone intake to develop rapport with parents, identify and address likely barriers to treatment, increase parental self-efficacy, hope and treatment motivation	Single time point	Not standardised – several weeks to months	Accessing care
32	Stevens, 2009/USA	179/179	11–20	Primary care	Adolescents who screened positive for at least one of depressive symptoms, suicidal ideation or substance abuse	Depression, suicide and substance abuse	Three phone calls in the first weeks after the first visit to the adolescent management clinic to assess youth's understanding of recommendations, address youth's struggles through case management and use motivational interviewing techniques if youth was ambivalent about treatment	Several weeks to months	6 months	Accessing care
33	Szapocznik, 1988/USA	108/108	12–21	Mental health centre	Adolescent drug abusers and their families	Substance abuse	Engagement intervention during intake interview to overcome family's resistance to treatment by identifying family patterns that interfere with entry into treatment	As many contacts as necessary within 3-week period	3 weeks	– Accessing care – Health outcomes
34	Wiseman, 1998/UK	128/128	Not stated	Child mental health clinic	Parents of prospective clients	Non-emergency general mental health problems	Didactic and interactive multi-family psycho-education group program; parent sessions focused on providing social support, information, skills, while children sessions focused on feeling less alone, understanding symptoms and effects of treatment, and building social skills	Single time point	Directly post-intervention	Accessing care

## Appendix 6

How outcomes were measured in each study

**Table:**

Universal school-based interventions targeting the general population									
	First author (year)	Intervention description	Outcomes studied						
			Knowledge	Attitudes	Intensions	Help-seeking	Action taken	Accessing care	Health outcomes
1	Aseltine (2007)	Two-part suicide prevention program: (1) curriculum via video with dramatisations, interviews and guidelines including discussion guide over half of the school year; (2) screening on depression and suicidality symptoms				Talking to an adult due to feeling depressed or suicidal		Receiving specialist care	Suicidal ideation (suicide attempts also measured as an outcome)
2	Campos (2018)	Interactive two-session educational curriculum that explored students' knowledge and beliefs about physical and mental health and illness, identified risk factors and symptoms of mental disorders as well as help-seeking options, promoted non-stigmatised behaviours and inadequate beliefs related to mental disorders and addressed self-help strategies and mental health-promoting behaviour	Knowledge about first aid skills and help-seeking						
3	Hart (2018)	Three standardised pyschoeducation sessions via program booklet, presentations, videos, role-plays, group and workbook activities, and final certificate for peer training to help adolescents with a mental health problem	Knowledge about when to recommend that another person seek help	Confidence in supporting a peer	Endorsing intentions to help a peer to seek help				
4	Howard (2018)	Two brief online educational interventions consisting of one page of a vignette about a person with depression: a biological condition describing the biological causes of depression and a psychosocial condition describing the psychosocial causes of depression			Help-seeking intentions				
5	Husky (2011)	Brief, two-stage mental health screening via questionnaire and structured interview; referrals to mental health care were provided for those who screened positive				Any student assistance contact		Any access to community-based services (any access to school-based services also measured)	
6	Jorm (2010)	Two day-long teacher training sessions on common mental health disorders, school policy on mental health issues and how to assist students in need	Beliefs about treatment for depression^a^	Confidence to talk with students about mental health problems^a^	Help-seeking intentions^b^		Spoke to students about mental health problems occasionally or more^a^		Mental health (abnormal SDQ score)
7	Klingman (1993)	Twelve weekly sessions delivering an educational curriculum on mental distress and disorders, help-seeking, and prevention via theoretical component, role-playing and rehearsing new skills	Knowledge of facts about help resources						
8	Morgan (2019)	A 14-h standardised pyschoeducation and training program for parents of adolescents to learn to recognise early signs of a mental health problem or crisis and to assist adolescents to access appropriate professional help as early as possible		Parental confidence to help adolescent^b^	Parental intensions to help adolescent^b^	Help-seeking by adolescent	Quality of parental support^b^		Adolescent mental health
9	Painter (2017)	Three-hour educational curriculum on stigma, mental illness and barriers to help-seeking via presentation, class discussion and video and presentation and discussion with a mentally ill college student	Knowledge about when to recommend that another person seek help	Personal willingness to seek help					
10	Perry (2014)	A 10 h of educational curriculum delivered over 5–8 weeks; resources for teachers delivering intervention included a booklet, slideshow and appendices on mental health, mood disorders and on helping others and oneself		Explicit attitudes toward seeking help					Psychological distress (suicidal ideation also measured as an outcome)
11	Saporito (2011)	35-min presentation on mental illness and its treatment plus video on an adolescent with a mental illness		Explicit attitudes toward seeking professional help					Emotional health (change in negative affect measured by PANAS)
12	Sayal (2010)	School-based screening to identify children with high ADHD rating scale scores and a book about ADHD and how to manage and work with affected children						Specialist service use	Symptoms of hyperactivity and inattention
13	Sharpe (2017)	Booklets on mental health and disorders and help-seeking for students				Frequency of seeking help from counsellor			Mental health (measured by Me and My School Questionnaire)

**Table:**

Interventions to engage individuals in health-care system								
	First author (year)	Intervention description	Outcomes studied					
			Knowledge	Attitudes	Help-seeking	Accessing care	Health outcomes	Satisfaction
14	Asarnow (2005)	A 6-month health service quality improvement intervention through support and training for clinicians on treatment for people with mental disorders by expert leader teams and care managers				Proportion of subjects who accessed any speciality mental health care	Depressive symptoms (measured by CES-D)	Satisfaction with mental health care
15	Asarnow (2011)	Brief family-based therapy session to increase motivation during emergency room visit by reframing the problem, educating families about the importance of treatment, obtaining commitment from youth, identifying triggers and developing and practising a safety plan supplemented by care linkage telephone contacts within the first 48 h after discharge				Outpatient mental health treatment	Suicide attempts	
16	Coker (2019)	A 5-min video on community mental health clinic and scheduled visit for a telehealth eligibility screening				Community mental health clinic screening visit	Health-related quality of life (measured by the Pediatric Quality of Life Inventory)	Satisfaction with the referral process
17	Donohue (1998)	Telephone call by clinical psychology doctoral students to parents about treatment, intake session for parent and youth, motivational reminder calls and incentives to participate in treatment				Proportion of subjects who attended the first appointment		
18	Fristad (2003)	Didactic and interactive multi-family psycho-education group program; parent sessions focused on providing social support, information and skills, while child sessions focused on feeling less alone, understanding symptoms and effects of treatment and building social skills		Perceived social support from parents		Ability to obtain appropriate services	Illness severity (measured by CDRS-R, MRS)	
19	Gadomski (2010)	Three hour-long communication skills training sessions for primary care clinicians to engage parents and children in diagnosis and treatment and address barriers to treatment with group discussions and 10-min practice visits				Number of primary care visits		
20	Grupp-Phelan (2012)	Discussion with a study social worker about screening results, patient concerns and available resources; designed to target various barriers and increase motivation for help-seeking behaviour			Proportion of subjects who scheduled the first appointment	Proportion of subjects who attended the first appointment	Depressive symptoms (measured by CES-D)	Screening found to be helpful
21	Gully (2008)	Educational booklet for parents on expectations and perceived value of treatment reviewed together with nurses	Knowledge about evidence-based treatment	Belief that evidence-based treatment is helpful		Proportion of subjects who attended the first appointment and discussed evidence-based treatment during appointment		Satisfaction with services
22	Kourany (1989)	Reminder telephone call, letter describing what would happen on the first clinic visit, or both the call and the letter				Proportion of subjects who attended the first appointment		
23	Lieberman (2006)	Provision of on-site mental health services (usual care was a referral to an off-site mental health provider)				Meeting with a counsellor at least once		
24	MacLean (1989)	One of four experimental letters (systematic appointment reminders, change slips requesting if appointment time should be changed, warnings and change slips combined with warnings)				Proportion of subjects who attended the first appointment		
25	McKay (1996*a*)	Intensive 30-minute telephone intervention with a social worker to engage caretaker in help-seeking process by identifying child's problem, framing caretaker actions as having potential to impact the situation and exploring barriers to help-seeking				Proportion of subjects who attended the first appointment		
26	McKay (1996*b*)	Telephone intake with therapists trained in specific engagement skills, i.e., informing clients about the process of obtaining mental health services, responding to concrete concerns or crisis situations and exploring potential barriers to obtaining services				Proportion of subjects who attended the first appointment		
27	McKay (1998)	Thirty-minute telephone and in-person engagement intervention by master's level clinicians to clarify the need for mental health care, increase the caretaker's investment in help-seeking, identify attitudes about and previous experiences with mental health care and over concrete barriers to accessing services				Proportion of subjects who attended the first appointment (proportion of sessions attended *v*. scheduled was also measured)		
28	Parrish (1986)	Letter informing parents that children would be moved to the bottom of the waiting list if three appointments were missed or letter informing parents that attending appointments would earn a coupon for winning a prize				Proportion of subjects who attended the first appointment		
29	Planos (1986)	Appointment reminder (telephone or letter prompt)				Proportion of subjects who attended screening appointments		
30	Richardson (2014)	A 12-month collaborative care intervention delivered by master's-level clinicians involving initial in-person education engagement session, choice of treatment and regular follow-up				Proportion of subjects with any specialty mental health visits according to administrative data	Depressive symptoms (measured by CDRS-R)	Satisfaction with treatment
31	Stern (2015)	A 10–15 min enhanced engagement phone intake to develop rapport with parents, identify and address likely barriers to treatment, increase parental self-efficacy, hope and treatment motivation				Attendance of first face-to-face interview		
32	Stevens (2009)	Three phone calls in the first weeks after the first visit to the adolescent management clinic to assess youth's understanding of recommendations, address youth's struggles through case management and use motivational interviewing techniques if youth was ambivalent about treatment				Any mental health service use		
33	Szapocznik (1988)	Engagement intervention during intake interview to overcome family's resistance to treatment by identifying family patterns that interfere with entry into treatment				Proportion of subjects visiting centre for intake appointment	Psychiatric and psychosocial functioning (measured by PSS)	
34	Wiseman (1998)	Didactic and interactive multi-family psycho-education group program; parent sessions focused on providing social support, information, skills, while children sessions focused on feeling less alone, understanding symptoms and effects of treatment and building social skills				Proportion of subjects who attended the first appointment		

^a^Teacher.

^b^Parent.
